# Tissue-specific transcriptome analyses reveal candidate genes for stilbene, flavonoid and anthraquinone biosynthesis in the medicinal plant *Polygonum cuspidatum*

**DOI:** 10.1186/s12864-021-07658-3

**Published:** 2021-05-17

**Authors:** Xiaowei Wang, Hongyan Hu, Zhijun Wu, Haili Fan, Guowei Wang, Tuanyao Chai, Hong Wang

**Affiliations:** 1 College of Life Sciences, University of Chinese Academy of Sciences, No.19(A) Yuquan Road, Shijingshan District Beijing, 100049 People’s Republic of China; 2 School of Life Sciences and Biotechnology, Heilongjiang Bayi Agricultural University, Daqing, 163319 China; 3 Institute of Genetics and Developmental Biology, Chinese Academy of Sciences, Beijing, 100101 China

**Keywords:** *Polygonum cuspidatum*, Transcriptome, Stilbenes, Flavonoids, Anthraquinones, Biosynthesis

## Abstract

**Background:**

*Polygonum cuspidatum* Sieb. et Zucc. is a well-known medicinal plant whose pharmacological effects derive mainly from its stilbenes, anthraquinones, and flavonoids. These compounds accumulate differentially in the root, stem, and leaf; however, the molecular basis of such tissue-specific accumulation remains poorly understood. Because tissue-specific accumulation of compounds is usually associated with tissue-specific expression of the related biosynthetic enzyme genes and regulators, we aimed to clarify and compare the transcripts expressed in different tissues of *P. cuspidatum* in this study.

**Results:**

High-throughput RNA sequencing was performed using three different tissues (the leaf, stem, and root) of *P. cuspidatum*. In total, 80,981 unigenes were obtained, of which 40,729 were annotated, and 21,235 differentially expressed genes were identified. Fifty-four candidate synthetase genes and 12 transcription factors associated with stilbene, flavonoid, and anthraquinone biosynthetic pathways were identified, and their expression levels in the three different tissues were analyzed. Phylogenetic analysis of polyketide synthase gene families revealed two novel *CHS* genes in *P. cuspidatum*. Most phenylpropanoid pathway genes were predominantly expressed in the root and stem, while methylerythritol 4-phosphate and isochorismate pathways for anthraquinone biosynthesis were dominant in the leaf. The expression patterns of synthase genes were almost in accordance with metabolite profiling in different tissues of *P. cuspidatum* as measured by high-performance liquid chromatography or ultraviolet spectrophotometry. All predicted transcription factors associated with regulation of the phenylpropanoid pathway were expressed at lower levels in the stem than in the leaf and root, but no consistent trend in their expression was observed between the leaf and the root.

**Conclusions:**

The molecular knowledge of key genes involved in the biosynthesis of *P. cuspidatum* stilbenes, flavonoids, and anthraquinones is poor. This study offers some novel insights into the biosynthetic regulation of bioactive compounds in different *P. cuspidatum* tissues and provides valuable resources for the potential metabolic engineering of this important medicinal plant.

**Supplementary Information:**

The online version contains supplementary material available at 10.1186/s12864-021-07658-3.

## Background

*Polygonum cuspidatum* Sieb. et Zucc., also known as Huzhang in China, Japanese knotweed in Japan, and Mexican bamboo in North America, is a herbaceous perennial of the Polygonaceae family [1]. It has been officially listed in the Chinese Pharmacopoeia and both its underground and above-ground parts have been widely used for centuries in the form of powder, extracts, and herbal infusions for the treatment of inflammatory diseases, infections, hyperlipidemia, and other disorders [2, 3]. For example, *P. cuspidatum* is listed as an important component of the recommended Chinese patent medicine Yinpian in the Diagnosis and Treatment Protocol for COVID-19 (Trial Version 7) [4].

The pharmacological effects of *P. cuspidatum* result from the presence of large amounts of bioactive compounds, including stilbenes, anthraquinones, and flavonoids [1]. For example, resveratrol and polydatin (glycosylated resveratrol), the most abundant stilbenes in *P. cuspidatum*, have provable curative effects in cancer, HIV, inflammation, and cardiovascular-related diseases [5]. Additionally, emodin and its derivative physcion, which are major anthraquinones in *P. cuspidatum*, have potential anticancer and antimicrobe applications [6, 7], while quercetin, catechin and their glycosides, major flavonoids in *P. cuspidatum*, have notable cardioprotective and anti-diabetic effects, promote the immune system, and protect the skin [8].

Although the pharmacological properties and chemical constituents of *P. cuspidatum* have been extensively studied, the biosynthetic pathways and regulatory mechanisms of its active compounds remain poorly understood because of limited molecular information. To our knowledge, few studies have investigated the molecular biology of *P. cuspidatum* in the literature. These include the publication of a draft genome (2.6 Gb) with a large number of scaffolds and assembly gaps, indicating that the *P. cuspidatum* genome contains multiple repeat sequences and high heterozygosity [9], two RNA sequencing transcriptomes from *P. cuspidatum* roots [10] and UV-C-treated leaves [11], and a report of the *Fagopyrum tataricum* genome (489.3 Mb) at the chromosome scale for the Polygonaceae family to which *P. cuspidatum* belongs [12]. This general lack of molecular knowledge on *P. cuspidatum* severely hampers an understanding of the biosynthesis mechanism of its active compounds.

Stilbenes, anthraquinones, and flavonoids accumulate differentially in the root, stem, and leaf of *P. cuspidatum* [1, 13]; however, the molecular mechanism underlying this tissue-specific accumulation is currently unclear. In general, the tissue-specific accumulation of compounds implies that related biosynthetic enzyme genes and regulators also have tissue-specific expression patterns; hence, tissue-specific transcriptome analysis is considered a promising method to reveal the regulatory mechanism of bioactive compound synthesis.

In this work, the transcripts expressed in different tissues of *P. cuspidatum* were investigated to clarify the molecular mechanism of the differential accumulation of stilbenes, anthraquinones, and flavonoids. More specifically, we performed the first known transcriptome profiling of three medicinal tissues (leaf, stem, and root) of *P. cuspidatum* using the HiSeq X Ten system. We identified synthase genes associated with the biosynthesis of stilbenes, anthraquinones, and flavonoids, and measured the relative expressions of these genes in different tissues. Our results offer novel insights into the biosynthetic regulation of bioactive compounds in different *P. cuspidatum* tissues from a molecular basis and provide valuable resources for the potential metabolic engineering of this important medicinal plant.

## Results and discussion

### Transcriptome sequencing and assembly

**Fig. 1:**
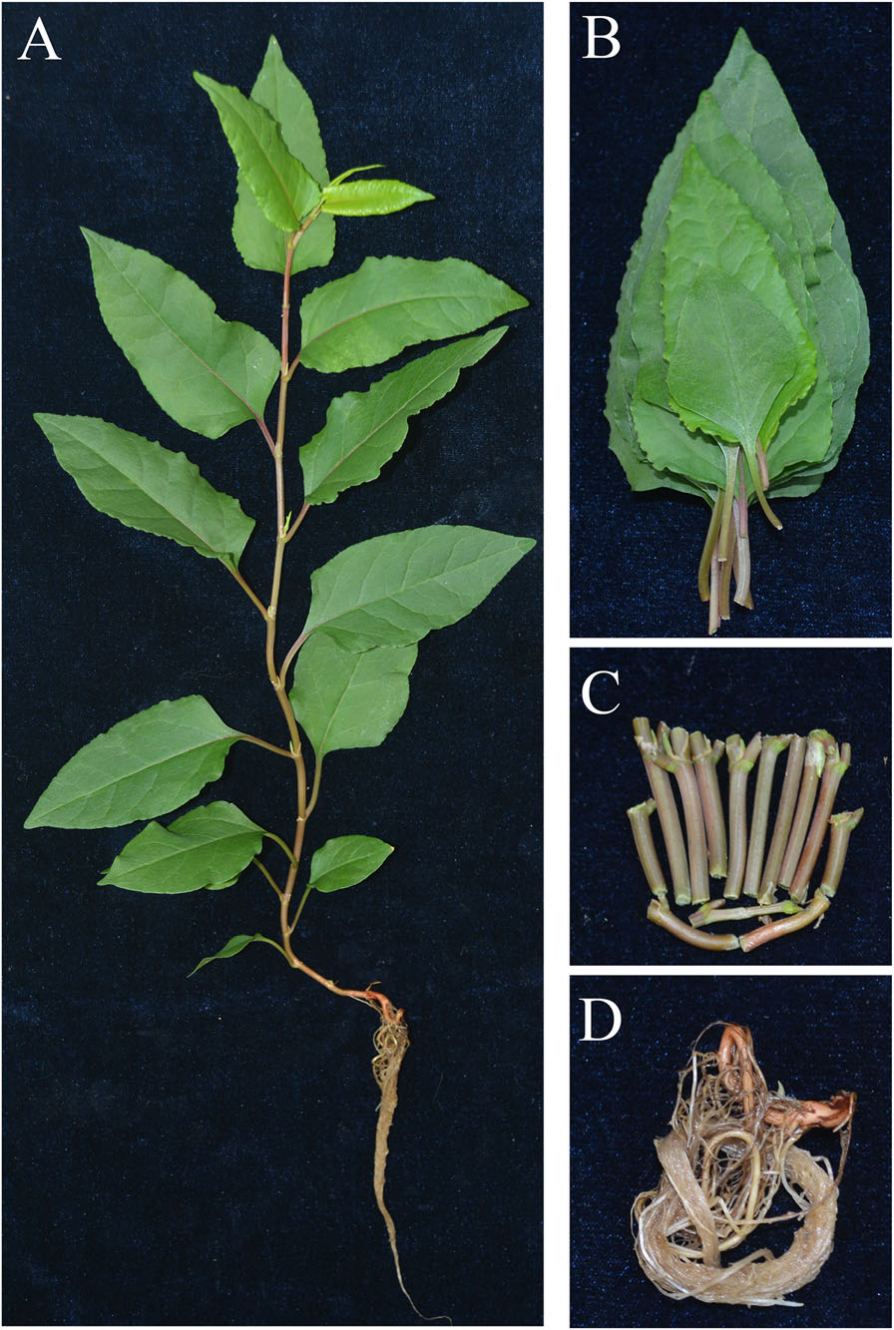
Plants and sampling of *Polygonum cuspidatum*. **a** Whole plant for sampling. **b** Leaf sample. **c** Stem sample. **d** Root sample

To construct a de novo transcriptome database, nine mRNA libraries prepared from leaf, stem, and root tissues of *P. cuspidatum* were sequenced using the Illumina HiSeq X Ten platform (Fig. 1). After filtering out low-quality reads, ∼24.2 million clean reads containing ∼72.53 Gb of clean nucleotides were obtained from all samples (Table 1). The Q30 value was more than 85.29%, and the GC content was 47.10–51.33% (Table S1). The combined assembly obtained 80,981 unigenes, of which 22,485 (27.77%) were longer than 1 kb (Additional File 1; Additional File 3:  Fig. S1). Their average length was 890 bp and the N50 length was 1440 bp (Table 1). The N50 value of the assembled data was similar to that recorded previously in other non-model plants, such as *Andrographis paniculata* [14] and *Isodon amethystoides* [15]. The completeness (complete single-copy BUSCOs 73.1% and complete duplicated BUSCOs 2.1%) of the assembly was assessed using BUSCO/v3.0.2 (Fig. S2). These data illustrate that the assembly results were favorable and applicable for subsequent studies. The average GC content of *P. cuspidatum* transcripts was in agreement with that reported previously for *P. cuspidatum* root (48.74%) [10], which is much higher than that of *Arabidopsis* (42.5%) and lower than that of rice (55%) [14].

**Table 1:** Summary of the transcriptome assembly of *Polygonum cuspidatum*

Sequences	Statistics
Clean reads	242,789,187
Clean nucleotides (nt)	72,529,952,472
GC percentage (%)	48.84%
Unigene number	80,981
Total length (nt)	72,065,482
Mean length (nt)	890
N50 (nt)	1440

### Functional annotation

The assembly was annotated using the Basic Local Alignment Search Tool (BLAST) (e < 10^− 5^) against NCBI non-redundant (Nr), Gene Ontology (GO), Clusters of Orthologous Groups of proteins (COGs), Swiss-Prot, EuKaryotic Orthologous Groups (KOG), Protein family (Pfam), eggNOG, and Encyclopedia of Genes and Genomes (KEGG) public databases. As shown in Table 2, 40,729 (50.29% of the 80,981) unigenes had annotated information, with 39,679 (45.9%), 23,356 (28.8%), 11,511 (14.2%), 28,284 (34.9%), 15,449 (19.1%), 23,827 (29.4%), 26,948 (33.3%), and 37,145 (45.9%) being annotated against Nr, GO, COG, Swiss-Prot, KEGG, KOG, Pfam, and eggNOG databases, respectively. Functions were predicted from the most similar annotated sequences in those databases (Table S2). The relatively low observed ratio may relate to the limited genetic information for the *Polygonum* genus of plants available in public databases. The unannotated unigenes belong to untranslated regions, or non-coding RNAs [16], or they may be unique to *P. cuspidatum* which would be a valuable resource for the discovery of novel genes.

**Table 2:** Functional annotation statistics of *Polygonum cuspidatum* unigenes

Databases	Annotated Number	Ratio
Annotated with Nr	39,679	49.00%
Annotated with GO	23,356	28.84%
Annotated with GOG	11,511	14.21%
Annotated with Swiss-Prot	28,284	34.93%
Annotated with KEGG	15,449	19.08%
Annotated with KOG	23,827	29.42%
Annotated with Pfam	26,948	33.28%
Annotated with eggNOG	37,145	45.87%
All annotated unigenes	40,729	50.29%

KEGG pathway analyses help increase the understanding of biological functions and the interactions of genes related to primary and secondary metabolites. Our KEGG pathway analysis revealed that 15,449 unigenes were successfully assigned to 130 pathways (Table S3). Among these, 20 KEGG pathways contained 705 unigenes associated with secondary metabolic processes. The cluster of “phenylpropanoid biosynthesis” (262 genes; ko00940) was predominant, followed by “flavonoid biosynthesis” (88 genes; ko00941) and “terpenoid backbone biosynthesis” (76 genes; ko00900). Additionally, 76 and 10 genes were assigned to “stilbenoid, diarylheptanoid, and gingerol biosynthesis” (ko00945) and “flavone and flavonol biosynthesis” (ko00944), respectively. These annotations could be helpful for further metabolic studies and for identifying genes involved in the secondary metabolism of *P. cuspidatum*. Finally, we identified several genes underlying stilbene, flavonoid, and anthraquinone synthesis by KEGG annotation homology analysis, including 28 genes in the phenylpropanoid biosynthetic pathway and 26 genes in the isochorismate, mevalonate (MVA), and methylerythritol 4-phosphate (MEP) pathways (Table S4). These genes could be of use for subsequent research into regulating the biosynthesis of stilbenes, flavonoids, and anthraquinones.

### Gene expression

**Fig. 2:**
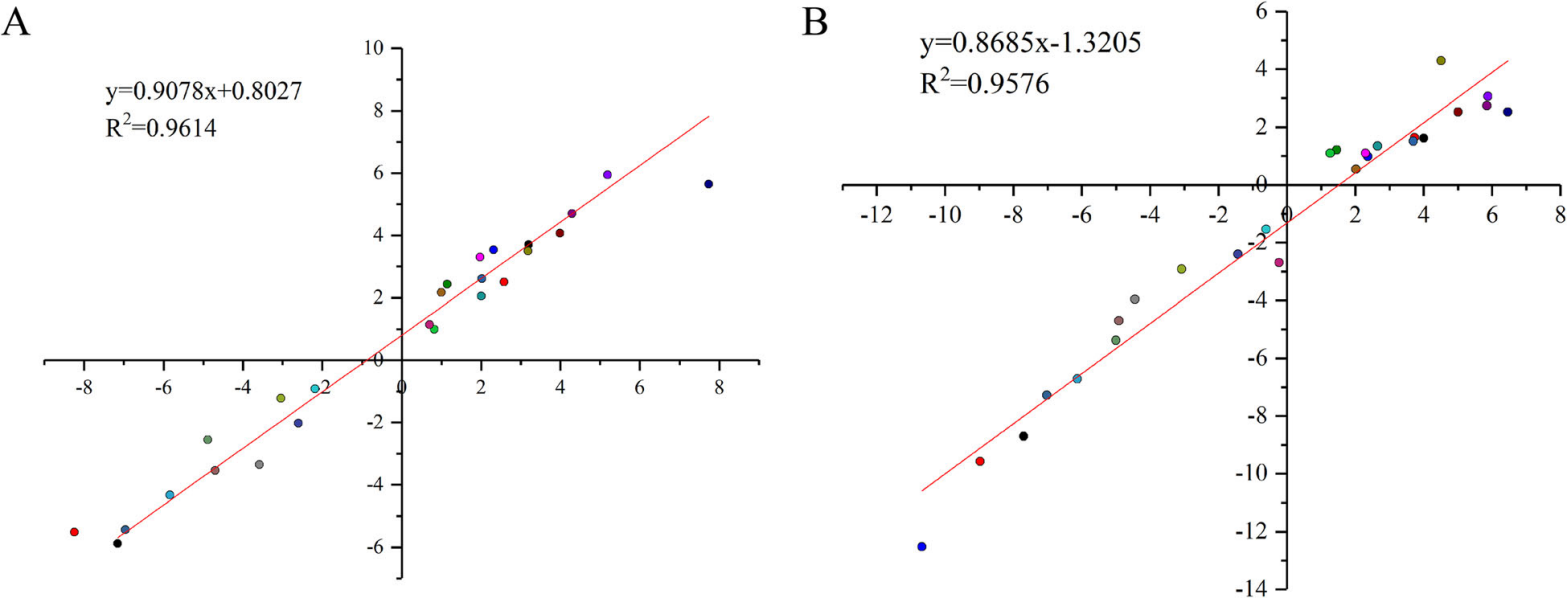
Correlation analysis of gene expression levels between real-time quantitative PCR (RT-qPCR) and transcriptome data for 25 selected *Polygonum cuspidatum* genes. **a** Each colored point represents an expression level-based fold-change value from the stem compared with a value from the leaf. **b** Each point represents an expression level-based fold-change value from the root compared with a value from the leaf

A total of 80,981 genes were expressed, with Fragments Per Kilobase of transcript per million mapped reads (FPKM) values ranging from 0.054 to 16,285.03, revealing high detection sensitivity (Table S5). Using a boxplot graph, differences in global gene expression levels in different samples were visually compared, and median expression quantities shown to occur in decreasing order from stem to leaf to root (Fig. S3A). Principal component analysis of the samples based on FPKM values showed that all biological replicates clustered together, which suggests the high reliability of our RNA-sequencing data (Fig. S3B). To further confirm the quality of our dataset, 25 genes differentially expressed in the leaf, stem, and root were selected for quantitative real-time RT-qPCR. Relative expression levels of all 25 genes determined from the transcriptome data were similar to those obtained in qPCR analysis. Positive correlations (R^2^ = 0.9614 and 0.9576) (Fig. 2) also confirmed the reliability of the RNA-sequencing gene expression-based calculations. Hence, RNA-sequencing was used for subsequent gene expression analyses in different tissues.

### Differentially expressed gene (DEG) expression

**Fig. 3:**
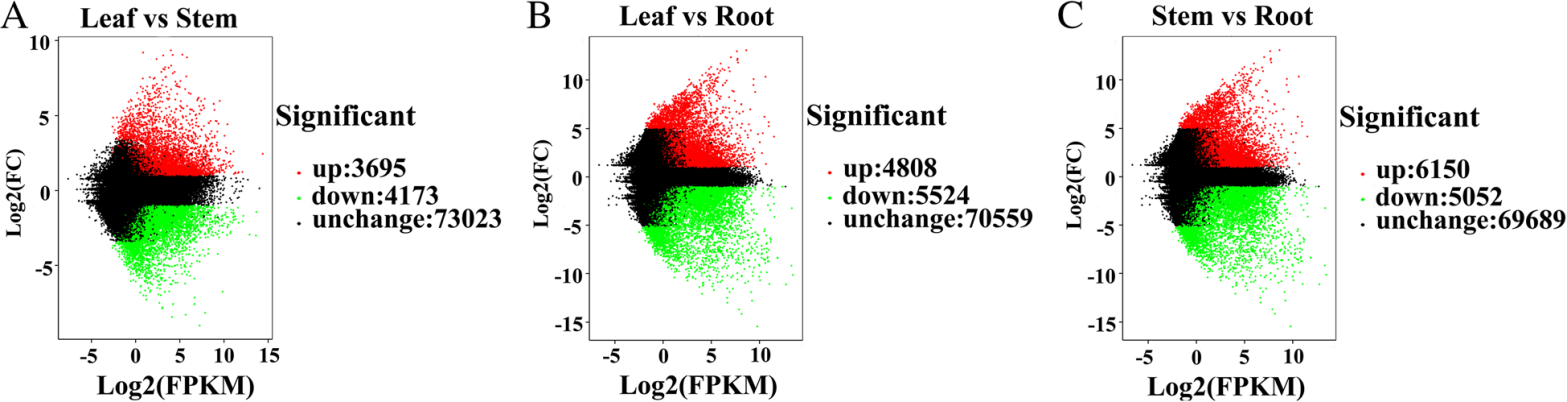
MA plots of transcriptome differences between different *Polygonum cuspidatum* tissues. **a** Leaf vs Stem, **b** Leaf vs Root, and **c** Stem vs Root. Differentially expressed genes (DEGs) of *Polygonum cuspidatum* were identified using the criteria FDR ≤0.01 and |log 2 (FC)| ≥ 1. Dark dots represent gene expression that was not significantly different in the comparisons, red dots represent up-regulated genes, and green dots represent down-regulated genes

**Fig. 4:**
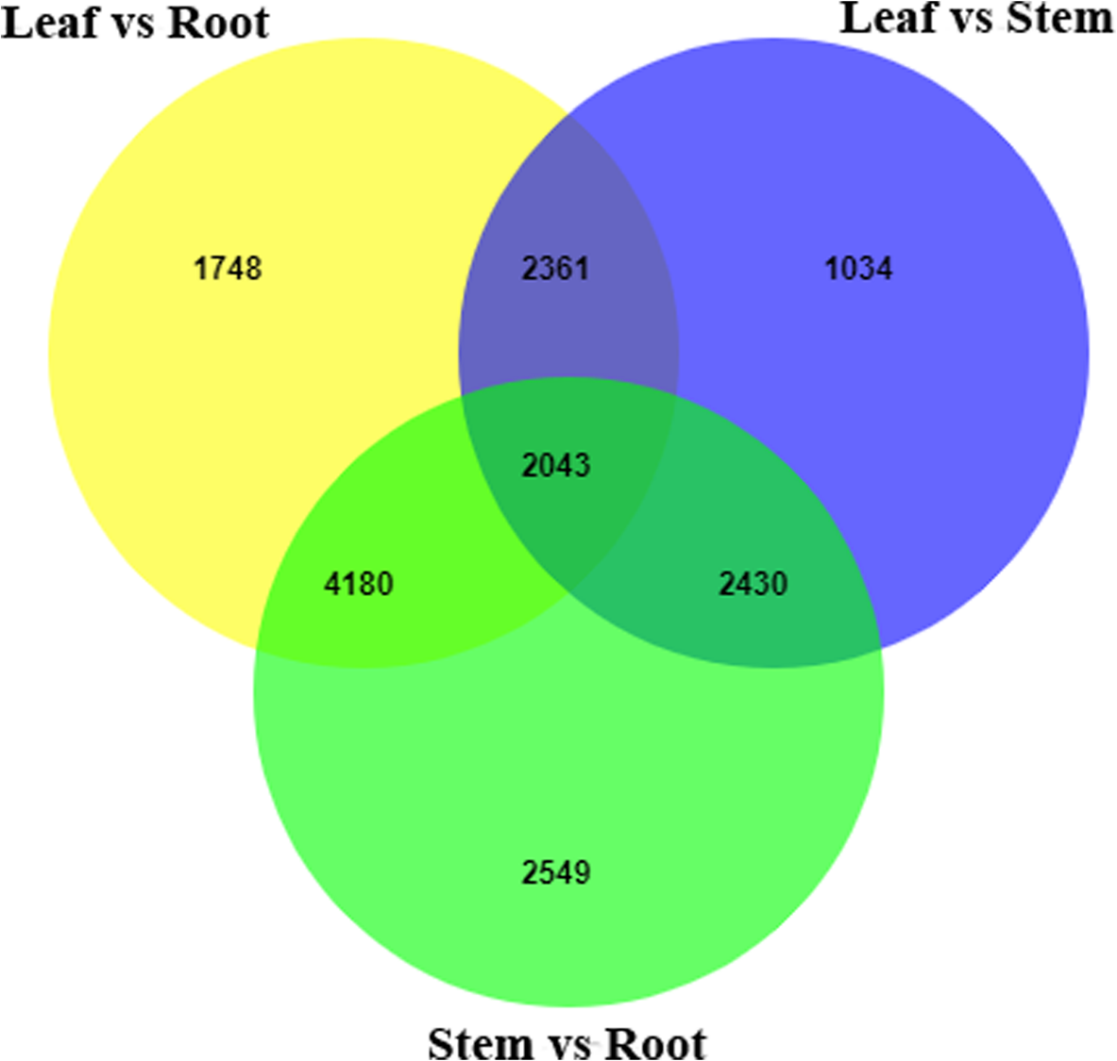
Venn diagram of DEGs identified in different *Polygonum cuspidatum* tissues. The common gene numbers are in overlapping regions by different comparisons

Thorough analyses of gene expression in different tissues under various conditions identified multiple DEGs, which provided a comparative landscape. Using the criteria of FPKM > 1, false discovery rate (FDR) < 0.01, and |log 2 (FC)| ≥1, 21,235 (26.22% of all unigenes) DEGs were identified. The pairwise comparisons of leaf vs stem, leaf vs root, and stem vs root revealed 7868 (4173 down-regulated, 3695 up-regulated), 10,332 (5524 down-regulated, 4808 up-regulated), and 11,202 (5052 down-regulated, 6150 up-regulated) DEGs, respectively (Fig. 3). Venn diagrams were constructed to illustrate the distributions and possible relationships of DEGs between paired comparisons, and 2043 DEGs were shown to be commonly altered (Fig. 4). To better understand the biological functions of these DEGs, GO and KEGG enrichment analyses were conducted. “Cell division”, “cell growth”, and “energy production”, which are related to basic plant functions, were shown to be enriched in pairwise comparisons. DEGs in the three tissues were also significantly enriched in “photosystem-related” terms as assessed by GO enrichment analysis (Table S6), as well as the “secondary metabolite biosynthetic process” term, indicating different metabolic and gene expression profiles for different tissues. For instance, differences in carotenoid biosynthesis in the leaf and root of *Daucus carota* [17] and flavonoid synthesis in the leaf, stem, and root of *Scutellaria viscidula* [18] represent typical examples of the tissue-specific biosynthesis of secondary metabolites. In our study, leaf vs stem and leaf vs root DEGs were enriched in GO terms related to lignin, flavonoid, and carotenoid metabolism; however, stem vs root DEGs were not enriched in these terms, revealing the close correlation between root and stem. Stem vs root DEGs were also enriched in “plant-type cell wall”, “chloroplast part”, “sulfate transmembrane transport”, “cation-transporting ATPase activity”, “2 iron, 2 sulfur cluster binding”, and “carbohydrate derivative transporter activity”, indicating the transport functions of the stem and photosynthetic characteristics (Table S6).

**Fig. 5:**
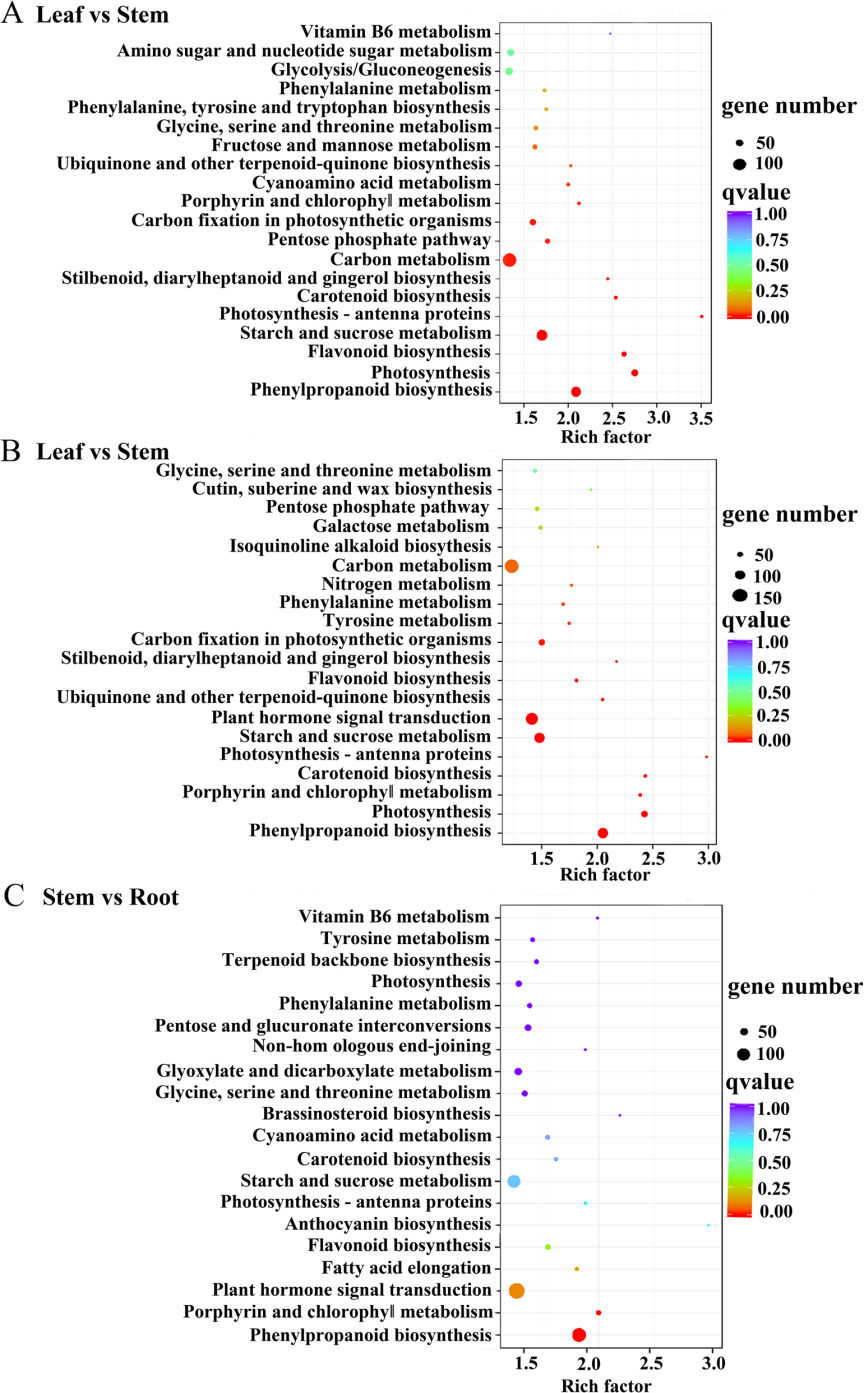
Scatterplot of differentially expressed *Polygonum cuspidatum* genes in the top 20 enriched KEGG pathways. **a** Leaf vs Stem, **b** Leaf vs Root, and **c** Stem vs Root. The y-axis shows the KEGG pathways, and the x-axis shows the enrichment factor. The enrichment factor is the ratio of the numbers of DEGs annotated in a certain pathway to the total number of genes mapped to this pathway. The *q*-value represents the corrected *P* value. A higher enrichment factor value correlates with a more intensive pathway, and a lower *q-*value with a more reliable one

The DEGs were enriched in 128 KEGG pathways, and the top 20 significant pathways in every pairwise comparison are listed in Fig. 5. The global maps of “phenylpropanoid biosynthesis”, “porphyrin and chlorophyll metabolism”, “flavonoid biosynthesis”, “photosynthesis-antenna proteins”, “starch and sucrose metabolism”, and “carotenoid biosynthesis” were all enriched in two of the three tissue comparisons. This indicated that genes related to these pathways were expressed in all three tissues but differed in their expression levels. In the stem vs root comparison, more DEGs were identified than in the other two comparisons, but there were fewer significantly enriched pathways (Fig. 5c).

### Stilbene and flavonoid biosynthesis in *P. cuspidatum*

**Fig. 6:**
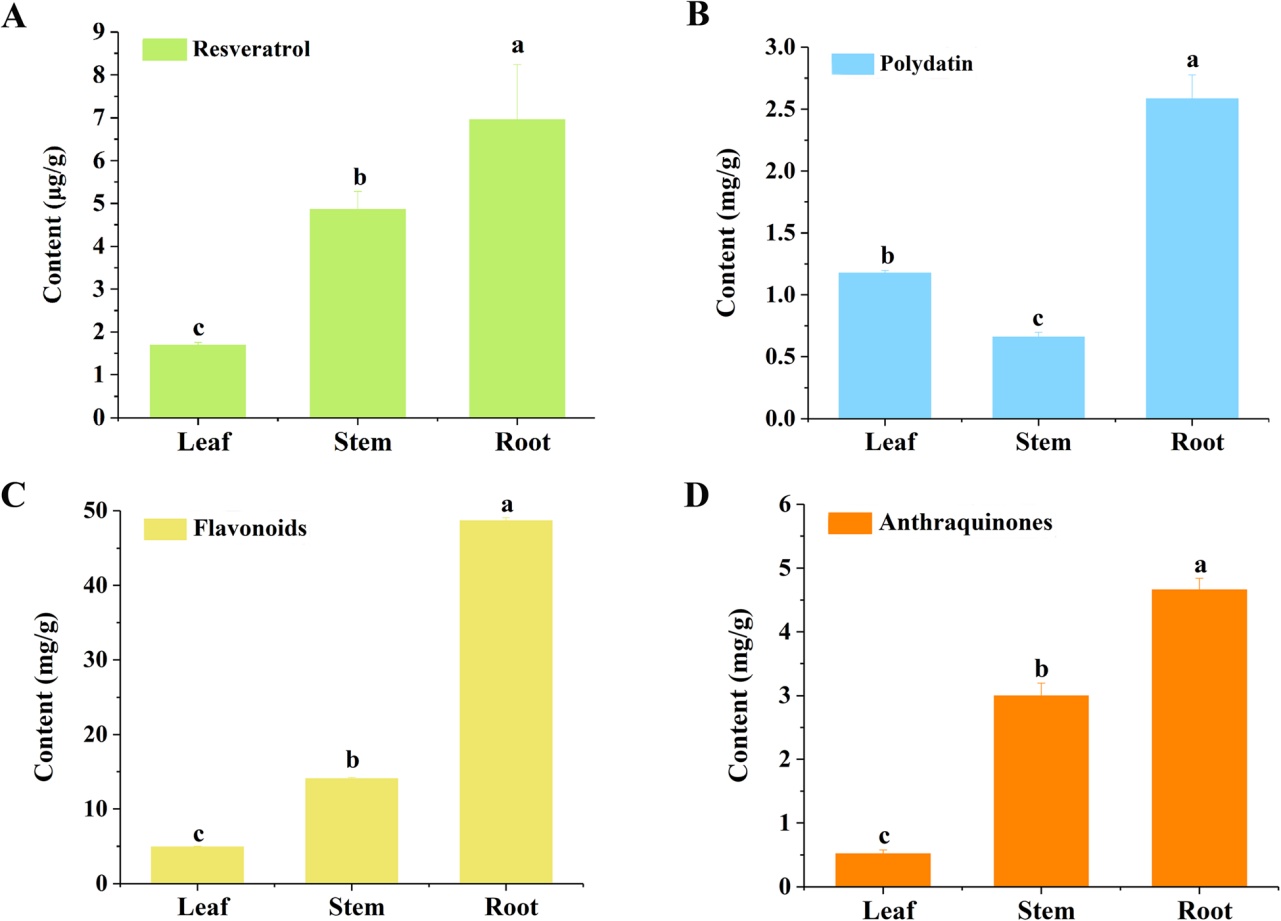
Quantification of metabolites in the leaf, stem, and root of *Polygonum cuspidatum* seedlings. **a** Resveratrol. **b** Polydatin. **c** Total flavonoids. **d** Total anthraquinones. Quantification of metabolites in the leaf, stem, and root of *Polygonum cuspidatum* seedlings. Values are expressed as the means ± standard errors of three independent samples. Significant differences (*p* < 0.05) were analyzed using a one-way ANOVA and indicated by lowercase letters **a**, **b**, and **c** in the leaf, stem, and root, respectively. Duncan’s multiple range test was used

Resveratrol and its derivatives polydatin (glycosylated product), pterostilbene (methylated product), and piceatannol (hydroxylated product) are the major stilbenes in *P. cuspidatum* [1]. The flavonoids are classified into flavones, flavanols, flavanones, flavanols, anthocyanins, isoflavones, and their derivatives on the basis of the saturation level, C-ring substitution pattern, and central pyrone C-ring opening [19]. To determine the contents of these two types of compounds and their distributions in different tissues, we measured stilbenes (resveratrol and polydatin) using high-performance liquid chromatography (HPLC) and total flavonoids using ultraviolet spectrophotometry. Resveratrol and polydatin were detected in all three tissues, while resveratrol and polydatin accumulated at higher levels in the root compared with the leaf and stem (Fig. 6a, b), in agreement with the common practice of using the root as the main medicinal *P. cuspidatum* tissue. The polydatin content of the root reached 2.59 ± 0.189 mg/g (dry weight; DW), meeting the requirement (≥0.15%) in the Chinese Pharmacopeia [2]. The resveratrol content ranged from 1.70 ± 0.057 to 6.97 ± 1.27 μg/g (DW) in different tissues, which was far lower than the values of ~ 0.1% in the *P. cuspidatum* root and ~ 0.002% in the leaf and stem reported by Yao et al. [20]. The total flavonoid content in the root (4.87%) was significantly higher than in the stem (1.41%) and leaf (0.49%) (Fig. 6c), which was consistent with the observed flavonoid tissue distribution in *S. viscidula* [18].

**Fig. 7:**
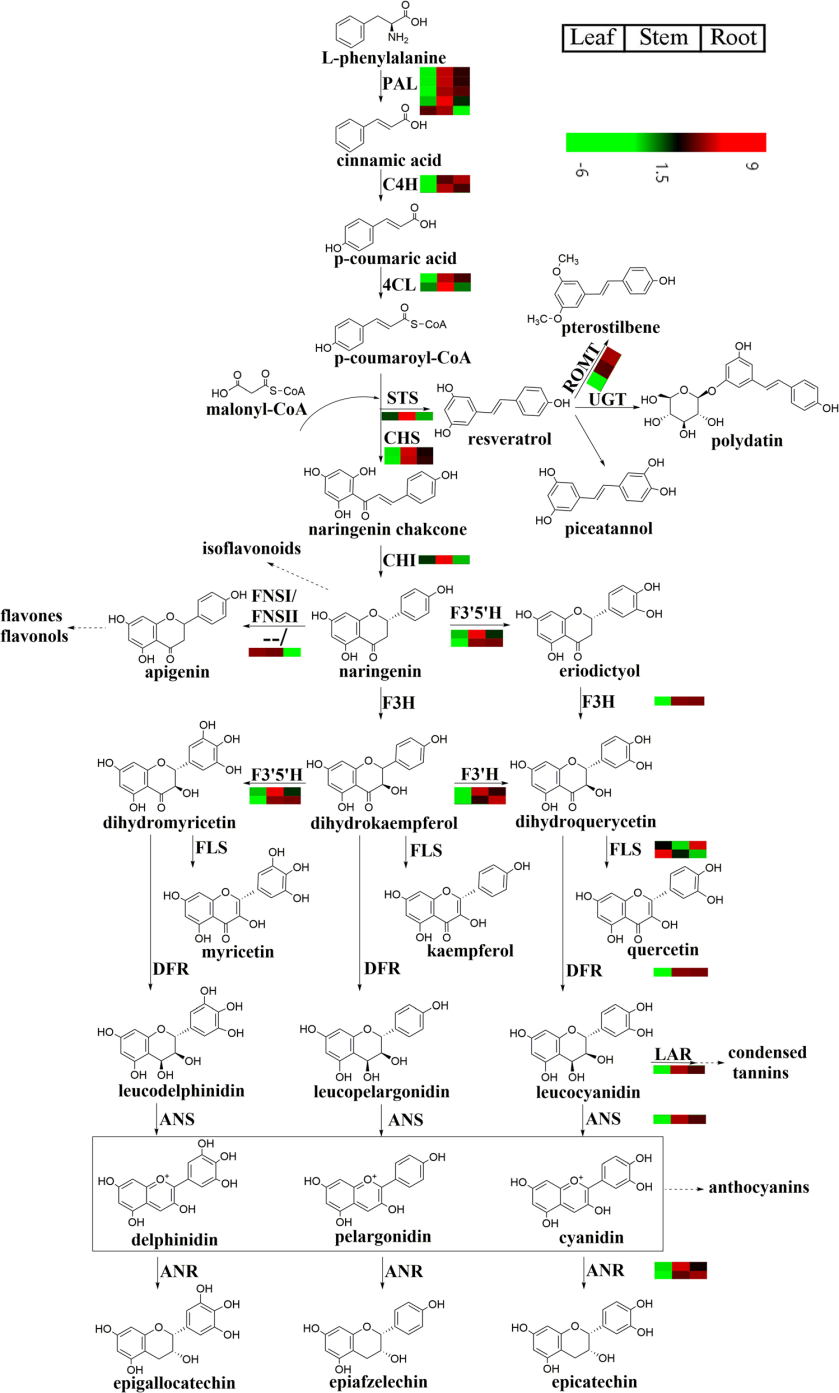
The phenylpropanoid pathway and the expression of related synthase genes in *Polygonum cuspidatum* tissues. The expression levels of unigenes encoding the enzymes of each step are shown. PAL, phenylalanine ammonia-lyase; C4H, trans-cinnamate 4- hydroxylase; 4CL, 4-coumarate-CoA ligase; CHS, chalcone synthase; CHI, chalcone isomerase; FNSI, flavone synthase I; FNS II, flavone synthase II; F3H, flavanone3-hydroxylase; F3′5’H, flavonoid 3′,5′-hydroxylase; F3′H, flavonoid 3′-hydroxylase; DFR, dihydroflavonol 4-reductase; FLS, flavonol synthase; ANS, anthocyanidin synthase; ANR, anthocyanidin reductase; STS, stilbene synthase; ROMT, trans-resveratrol di-O-methyltransferase; UGT, glycosyl transferase; LAR: leucoanthocyantin reductase. The phenylpropanoid metabolism pathway was drawn using ChemDraw software according to map 00940, map 00941 and map 00945 in KEGG pathway database; the copyright permission of use and modification of the above pathways has been granted

Although the phenylpropanoid pathway is well characterized in some plant species, such as *Arabidopsis*, grape, and petunia [21], limited information is available for *P. cuspidatum*. To investigate the molecular bases for stilbene and flavonoid biosynthesis in this plant, genes involved in this pathway were selected from the *P. cuspidatum* transcriptome, and their expression patterns were compared among leaf, stem, and root tissues. A total of 28 candidate genes for 16 enzymes of the phenylpropanoid pathway were identified (Table S4). The tissue-specific expression patterns of the synthase genes involved in the phenylpropanoid pathway are presented in a heat map (Fig. 7).

L-Phenylalanine is converted to cinnamic acid by the catalytic reaction of phenylalanine ammonia-lyase (PAL), which is the first committed step of the phenylpropanoid pathway. Then, cinnamate 4-hydroxylase (C4H) converts cinnamic acid to *p*-coumaric acid, which leads to the formation of *p*-coumaroyl-CoA by the action of 4-coumaroyl CoA-ligase (4CL) (Fig. 7). *PAL*s are encoded by a multigene family in plants [22], and five unigenes were identified as *PAL*s in our study. All *PAL*s, except *PAL5*, were expressed at high levels in the stem, at intermediate levels in the root, and at low levels in the leaf. *C4H* and *4CL* had high expression levels in the root and stem but lower levels in the leaf (Fig. 7). In *Allium sativum* [23] and *Polygonum minus* [24], the highest expression of *C4H* occurred in the root, while the expression of *4CL* in different organs differed among species [25, 26].

The chalcone synthase (*CHS*) gene encodes the first key enzyme in the flavonoid pathway that catalyzes the condensation of 1 *p*-coumaroyl-CoA molecule and three malonyl-CoA molecules to naringenin chalcone. The same substrates are used by resveratrol synthase (STS) to produce resveratrol, which represents the entrance to the stilbene pathway. Later, naringenin chalcone is isomerized by chalcone isomerase (CHI) to yield flavanone, and resveratrol is converted into pterostilbene by resveratrol O-methyltransferase (ROMT) and polydatin by UDP-glycosyltransferase (Fig. 7). We successfully identified two *CHS*, one *CHI*, one *STS*, and two *ROMT* genes in our *P. cuspidatum* transcriptome. One CHS protein sequence (named PcCHS3) shared 97.70 and 94.40% homology with PcCHS1 and PcCHS2 reported previously in *P. cuspidatum*, respectively; the other (named PcCHS4) had 93.62 and 92.62% similarity with PcCHS1 and PcCHS2, respectively [27, 28]. The sequence of *STS* was consistent with *PcSTS* previously cloned by our laboratory [28].

*STS* genes usually form small families of two to five closely related paralogs but have expanded into a super family of 48 members in *Vitis vinifera* [29]. Phylogenetic analysis was performed to compare *STS* and *CHS* genes with other polyketide synthase genes in *P. cuspidatum* and other species (Fig. S4). We observed a marked discrepancy between *STS* and *CHS* families, and *P. cuspidatum STS* sequences were found to be more closely related to those of *Rheum tataricum* and *Fallopia multiflora* than other resveratrol-producing plants. As presented in Table S4, the average FPKM value of the *CHS*s was approximately 20 times greater than that of the *STS*s. Two *CHS* genes exhibited higher expression levels in the stem and root than in the leaf. High expression of *STS* occurred in the leaf and stem, while the root showed the lowest expression level. *CHI* exhibited lower expression levels in the root than in the stem and leaf, which was consistent with the *CHI* expression pattern seen in *S. viscidula* [18]. Conversely, the expression level of *ROMT* in the root was significantly higher than in other tissues, which suggests that its active methylation occurred in the root.

Downstream of the phenylpropanoid pathway, flavone synthases (FNS) I and II catalyze the synthesis of apigenin from naringenin and then to flavones and flavonols through a series of enzymatic reactions. *FNSII* was shown to be highly expressed in the *P. cuspidatum* leaf, in line with the previously observed highest expression of its related products, apigenin-7-O-glucoside and apigenin-7-O-(6″-O-acetylglucoside), in the same tissue [30]. Naringenin is continually hydroxylated into eriodictyol, dihydrokaempferol, dihydroquercetin, or dihydromyricetin by flavanone 3-hydroxylase (F3H), flavonoid 3′-hydroxylase (F3′H), or flavonoid 3′,5′-hydroxylase (F3′5′H) (Fig. 7). Subsequently, several key enzymes, dihydroflavonol 4-reductase (DFR), anthocyanidin synthase (ANS), and anthocyanidin reductase (ANR), are involved in the biosynthesis of flavanol (epicatechin, epiafzelechin, and epigallocatechin) or anthocyanin (cyanidin, pelargonidin, and delphinidin). A branched pathway of condensed tannin synthesis originates from the intermediate products of anthocyanin catalyzed by leucoanthocyantin reductase (LAR). We identified two transcripts each for *F3′H*, *F3′5′H*, and *ANR*, which were all expressed at their lowest levels in the leaf, but the expression profiles of different transcripts for each synthetase differed between the stem and the root. The expression patterns of *F3H*, *DFR*, *LAR*, and *ANS* were similar, with higher expression in the root and stem than in the leaf. Because tender leaves and stems contain more tannins, especially in the spring and fall [3], the consumption of tender leaves and stems of *P. cuspidatum* should be moderated.

Flavonol synthase (FLS) is involved in the synthesis of flavonols such as quercetin, kaempferol, and myricetin (Fig. 7). We found two isoform *FLS* genes in *P. cuspidatum* which showed different expression levels in different tissues, which was consistent with the observed *FLS* expression in *Fagopyrum tataricum* [31]. The expression of *FLS1* was highest in the root and lowest in the leaf, while that of *FLS2* was highest in the leaf and lowest in the root. In our previous metabolic profiles of different *P. cuspidatum* tissues, quercetin xyloside and quercetin 4-glucoside were highly enriched in the leaf [30], which may result from the expression of *FLS2*.

### Anthraquinone biosynthesis in *P. cuspidatum*

Anthraquinone compounds, having the fundamental parental nucleus of an anthraquinone ring, are of interest owing to their effectiveness in treating constipation, arthritis, multiple sclerosis, and cancer [32]. Anthraquinones can be generally categorized into emodin- and alizarin-types based on their hydroxyl location either on both sides or on one side of the benzene ring, respectively [32]. For example, emodin, rhein, physcion, chrysophanol, and aloe-emodin belong to the emodin-type, while alizarin, purpurin, and pseudopurpurin belong to the alizarin-type. The total anthraquinone contents were evaluated in our study (Fig. 6d) and significant differences were observed among root (4.67 ± 0.18 mg/g), stem (3.00 ± 0.19 mg/g), and leaf (0.53 ± 0.054 mg/g) tissues. Yao et al. [20] also reported a high total anthraquinone content (~ 2%) in the annual root (~ 1.2%) and a low content in both the stem (~ 0.3%) and leaf (~ 0.2%). The low anthraquinone content in the present study may result from various factors, such as using the young seedling stage of *P. cuspidatum* or the greenhouse environment. In our previous metabolome analysis, the highest emodin, acetylemodin, aloe-emodin, and physcion contents were also found in the root [30]. Consequently, we speculate that anthraquinones largely accumulate in the root of *P. cuspidatum*.

**Fig. 8:**
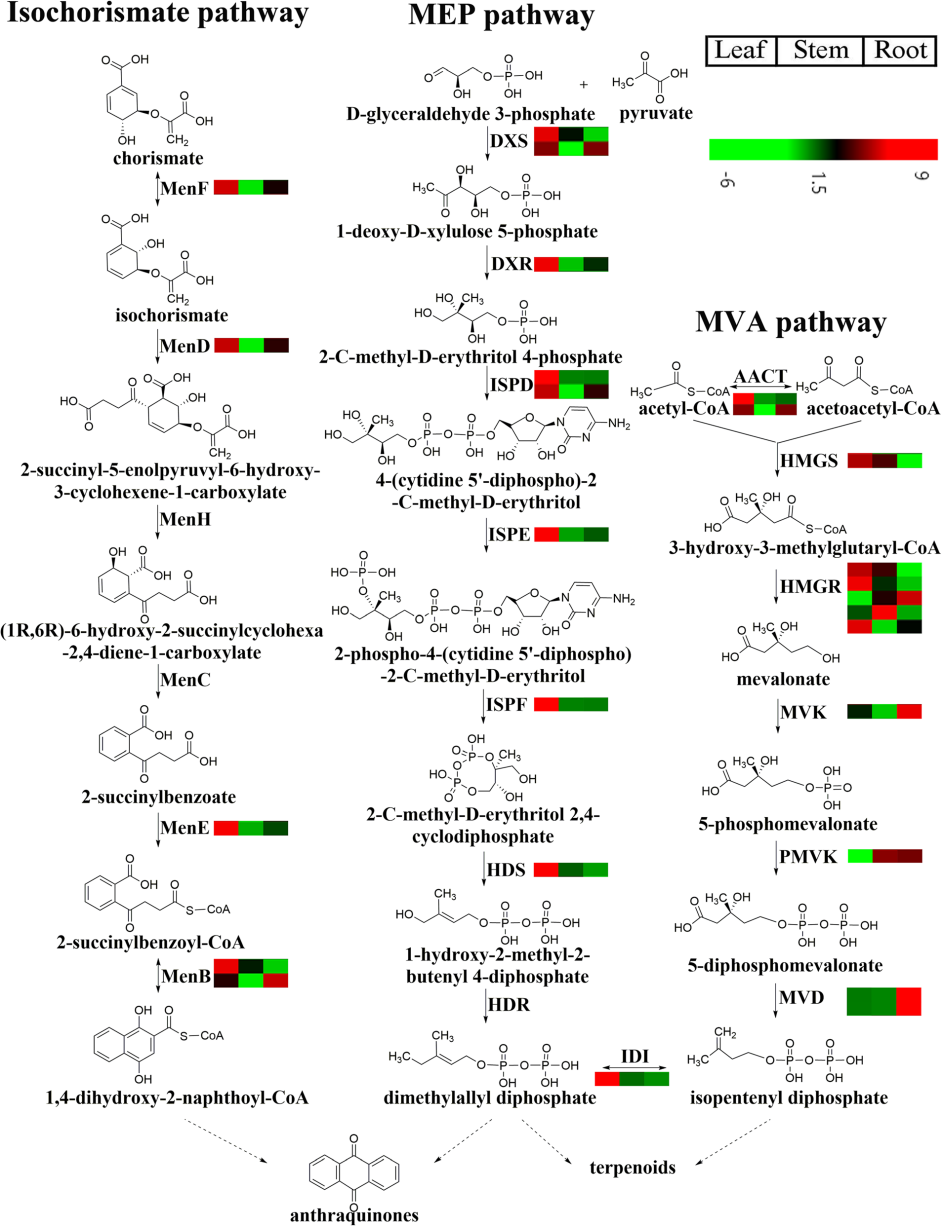
Plausible biosynthetic pathway and expression of unigenes involved in the biosynthesis of anthraquinones in *Polygonum cuspidatum*. The expression levels of unigenes encoding the enzymes of each step are shown. MenF, isochorismate synthase; MenD, 2-succinyl-5-enolpyruvyl-6-hydroxy-3-cyclohexene-1-carboxylate synthase; MenH, 2-succinyl-6-hydroxy-2,4-cyclohexadiene-1-carboxylate synthase; MenC, O-succinylbenzoate synthase; MenE, O-succinylbenzoic acid-CoA ligase; MenB, naphthoate synthase; DXS: 1-deoxy-D-xylulose-5-phosphate synthase; DXR: 1-deoxy-D-xylulose-5-phosphate reductoisomerase; ISPD, 2-C-methyl-D-erythritol 4-phosphate cytidylyltransferase; ISPE, 4-diphosphocytidyl-2-C-methyl-D-erythritol kinase; ISPF, 2-C-methyl-D-erythritol 2,4-cyclodiphosphate synthase; HDS, (E)-4-hydroxy-3-methylbut-2-enyl diphosphate synthase; HDR, 4-hydroxy-3-methylbut-2-enyl diphosphate reductase. AACT, acetyl-CoA C-acetyltransferase; HMGS, hydroxymethylglutaryl-CoA synthase; HMGR, hydroxymethylglutaryl-CoA reductase; MVK, mevalonate kinase; PMVK, phosphomevalonate kinase; MVD, diphosphomevalonate decarboxylase; IDI, isopentenyl-diphosphate delta-isomerase. The anthraquinone metabolism pathway was drawn using ChemDraw software according to map 00900 and map 00130 in KEGG pathway database; the copyright permission of use and modification of the above pathways has been granted

Limited information is available on the biosynthesis of anthraquinones in plants, and, to our knowledge, there are just two reports on anthraquinone synthesis-related enzymes in *P. cuspidatum* [9, 10]. The anthraquinone skeleton is formed by coupling the isochorismate pathway-derived 1,4-dihydroxy-2-naphthoyl-CoA with the MVA/MEP pathway-derived dimethylallyl diphosphate [33]. A deduced anthraquinone metabolic pathway was constructed for *P. cuspidatum* (Fig. 8). Using our functional annotation data and homologous alignment results, we identified 26 unigenes annotated as 18 enzymes involved in formation of the anthraquinone backbone, including 13 unigenes for seven enzymes involved in the MVA pathway, eight unigenes for seven enzymes involved in the MEP pathway, and five unigenes for four enzymes (except Men C and Men H) involved in the isochorismate pathway (Table S4). Most genes encoding enzymes involved in the isochorismate and MVA/MEP pathways were present in the transcriptome of *P. cuspidatum* in our study. 2-Succinylbenzoate was formed from isochorismate through a series of enzymatic reactions, but only one enzyme (Men D) was identified in our transcriptome data, similar to the findings in *Cassia angustifolia* [34] and *Senna tora* [35].

In the MVA pathway, isopentenyl-diphosphate delta-isomerase (IDI), which catalyzes the formation of isopentenyl diphosphate and its isomer dimethylallyl diphosphate, had the highest FPKM value. Mevalonate kinase and phosphomevalonate kinase, which may be involved in the rate-limiting steps of the MVA pathway, had the lowest FPKM values. Mevalonate kinase (MVK), phosphomevalonate kinase (PMVK), and diphosphomevalonate decarboxylase (MVD) were highly expressed in the root, while hydroxymethylglutaryl-CoA synthase and IDI were highly expressed in the leaf. Interestingly, five and two unigenes were found for hydroxymethylglutaryl-CoA reductase and acetyl-CoA C-acetyltransferase, respectively, and each showed different expression patterns in different tissues (Fig. 8). In the MEP pathway, all genes were significantly highly expressed in the leaf compared with the stem and root.

P-Deoxy-D-xylulose-5-phosphate synthase (DXS) is the first enzyme of the MEP pathway, and two unigenes were found for DXS that shared a 54.46% amino acid sequence identity but had different expression patterns. The expression levels of DXS, 2-C-methyl-D-erythritol 2,4-cyclodiphosphate synthase, I-4-hydroxy-3-methylbut-2-enyl diphosphate synthase, and 4-hydroxy-3-methylbut-2-enyl diphosphate reductase in the leaf were more than 10 times greater than in the root and 3–9 times greater than in the stem (Table S4). Our observed expression patterns of *MVK*, *PMVK*, *DXS*, and *DXR* in *P. cuspidatum* were consistent with those in *Aloe vera* [36]. Isochorismate is a product of the shikimate pathway. The unigenes encoding isochorismate synthase, 2-succinyl-5-enolpyruvyl-6-hydroxy-3-cyclohexene-1- carboxylate synthase, and O-succinylbenzoic acid-CoA ligase had low FPKM values (Table S4), and their expression levels were highest in the leaf, followed by the root then the stem. The expression patterns of two isoforms of naphthoate synthase followed opposite trends in different tissues (Fig. 8).

Thus, we conclude that MEP and isochorismate pathways for anthraquinone biosynthesis were dominant in the leaf tissue in our study. The sterols and terpenoids biosynthesis also shares the MEP pathway; however, how the distribution of intermediate metabolites is regulated needs further study. Anthraquinones may also be biosynthesized through the polyketide pathway in plants [37]. Polyketide synthase III and polyketide cyclase are key enzymes involved in the polyketide pathway, and the involvement of polyketide synthase III and UDP-glycosyltransferase in anthraquinone formation has been investigated [37–39]; however, the pathway details remain unclear. To date, no genes involved in the late steps of anthraquinone biosynthesis have been identified from *P. cuspidatum* or other anthraquinone-producing plants. Cytochrome P450s, methyltransferases, glucosyltransferases, and other enzymes involved in this process should therefore be studied comprehensively in the future.

### Transcription factors (TFs)

Increasing evidence suggests that various transcription factor (TF) families participate in the biosynthesis of secondary metabolites in plants [40]. The most abundant families annotated in *P. cuspidatum* include AP2 (107 genes), followed by bHLH (105 genes), GRAS (84 genes), C2H2 (82 genes), MYB-related (79 genes), WRKY (77 genes), B3 (77 genes), bZIP (77 genes), C2C2 (74 genes), MYB (66 genes), and C3H (66 genes) (Table S7). In total, 810 (57.94%) differentially expressed TFs were identified in this study, and bHLH, AP2, WRKY, C2H2, and bZIP families had the most differentially expressed TFs. The root had more up- than down-regulated TFs compared with the leaf and stem (Fig. S5).

According to previous reports, *STS* is activated by MYB15 and repressed by WRKY8 in *V. vinifera* [41]; flavonol biosynthesis is increased by GtMYBP4 [42], MtWRKY100630, and MtWRKY108715 [43], and reduced by FtMYB16 [44], FtMYB14 [44], and GmMYB100 [45]; and anthocyanin biosynthesis is negatively regulated by VvMYB4-like [46] and positively regulated by MdWRKY11 and MdWRKY40 [47]. We identified 12 homologous *MYB* and *WRKY* genes and constructed a heat map of their expression levels (Fig. S6). The lowest expression levels of all TFs occurred in the stem, and the highest expression levels of all *WRKY*s (*c75759.graph_c0*, *c88656.graph_c0*, *c89819.graph_c1*, *c79974.graph_c1*, and *c92547.graph_c0*) enhancing anthocyanin or flavonol synthesis were in the root. *MYB*-activating *STS* (*c86302.graph_c0*) was expressed at higher levels in the root than in the leaf, while *WRKY*-repressing *STS* (*c79443.graph_c0*) was expressed at higher levels in the leaf than in the root. The *MYB*s associated with flavone and flavonol synthesis showed different expression patterns in the leaf and root. The highest expression levels of two *MYB*s (*c72476.graph_c0* and *c79733.graph_c0*) that reduce rutin synthesis and one *MYB* (*c78111.graph_c0*) that increases flavonol biosynthesis were seen in the leaf, while the highest expression levels of two *MYB* TFs (*c37240.graph_c0* and *c83560.graph_c0*) that reduce flavonoid biosynthesis occurred in the root. Data mining and experimental verification are required to fully understand the complex transcriptional regulatory networks. The TFs identified in the *P. cuspidatum* database may play crucial roles in modifying the levels and increasing the production of secondary metabolites, and in reducing the level of undesirable metabolites that have adverse effects on the quality of *P. cuspidatum*.

## Conclusions

*P. cuspidatum* is a famous medicinal and edible plant that is desirable owing to its variety of stilbene-, flavonoid-, and anthraquinone-related bioactive compounds. To the best of our knowledge, we generated the first comprehensive transcriptome assembly of *P. cuspidatum* from leaf, stem, and root tissues in the present study. In total, 80,981 unigenes were obtained, 40,729 (50.29%) were annotated, and 21,235 (26.22%) DEGs were identified. The N50 of 1440 bases, principal component analysis-based classification, and RT-qPCR consistency confirmed the reliability of our RNA-sequencing data. “Metabolism” was the most abundantly enriched class for root-specific genes, and multiple DEGs mapped to secondary metabolic pathways including “phenylpropanoid biosynthesis”, “flavonoid biosynthesis”, “carotenoid biosynthesis”, “stilbenoid, diarylheptanoid, and gingerol biosynthesis”, and “ubiquinone and other terpenoid-quinone biosynthesis”.

A total of 1398 putative TFs genes were identified, and 57 promising candidate synthase genes, as well as six MYB and six WRKY regulators related to stilbene, flavonoid, and anthraquinone synthesis, were comprehensively analyzed. The phylogenetic trees of polyketide synthase genes showed two novel *CHS* genes in *P. cuspidatum*. Most genes (*PAL*, *C4H*, *4CL*, *CHS*, *ROMT*, *F3H*, *F3′5′H*, *DFR*, *LAR*, and *ANR*) involved in the phenylpropanoid pathway were highly expressed in the root and stem; however, *CHI*, *STS*, *FLS*, and *FNSII* were highly expressed in the leaf and stem. Additionally, MEP and isochorismate pathways for anthraquinone biosynthesis were dominant in the leaf. The expression patterns of synthase genes were almost consistent with the metabolite profiles in different tissues of *P. cuspidatum*. The lowest levels of all predicted TFs related to regulation of the phenylpropanoid pathway were seen in the stem and different expression patterns were exhibited in the leaf and root. Thus, we conclude that relatively abundant bioactive compounds are present in the leaf and stem, as well as in the root, providing a scientific basis for the maximum utilization of *P. cuspidatum*. Our results not only advance our understanding of the molecular basis of secondary metabolite biosynthesis in *P. cuspidatum*, but also provide valuable resources for future genetics and metabolic biology studies.

## Materials and methods

### Plant materials

*Polygonum cuspidatum* Sieb. et Zucc. was identified by Professor Xiangyun Zhu of the Institute of Botany, the Chinese Academy of Sciences. The voucher specimen (xyz2021001) of the plant is deposited at Herbarium (PE), Institute of Botany, the Chinese Academy of Sciences, Beijing, China. Seeds of *P. cuspidatum* were collected from the medicinal plant garden of the Institute of Botany, Chinese Academy of Sciences (Beijing, China), then surface-sterilized and sown on Murashige and Skoog agar medium in a climate chamber at a temperature of 24 ± 2 °C with a light/dark cycle of 16 h/8 h. One month later, the same seedlings were transplanted in plastic pots (8 × 8 × 8 cm) containing a soil/vermiculite mixture (1:1). The germination environmental conditions were 24–26 °C and 60–70% relative humidity. The plants were watered with 1‰ fertilizer every 4 days. Plant materials were excised from 3-month-old young seedlings and dissected into root, stem, and leaf (Fig. 1). Three biological replicates for each sample and three seedlings for every replicate were prepared. All samples were washed thoroughly with sterile water and immediately frozen in liquid nitrogen, then stored at − 80 °C and/or freeze-dried for RNA isolation and/or secondary metabolite analysis.

### RNA extraction and quality assessment

Total RNA was extracted from samples using the Plant Total RNA Purification Kit (GeneMark, Taiwan, China) following the manufacturer’s instructions. RNA degradation and contamination were monitored on a 1% (m/v) agarose gel. RNA purity and integrity were assessed using a NanoPhotometer® spectrophotometer (Implen, Westlake Village, CA, USA) and RNA Nano 6000 Assay Kit of the Agilent Bioanalyzer 2100 system (Agilent Technologies, Santa Clara, CA, USA), respectively. The RNA concentration was measured using a Qubit® RNA Assay Kit with a Qubit®2.0 Fluorometer (Life Technologies, Carlsbad, CA, USA). All RNA samples were within suitable parameters and were shipped on dry ice to the Biomarker Biotechnology Corporation (Beijing, China) for cDNA library construction and RNA sequencing.

### cDNA library construction and sequencing

Nine libraries (3 different tissues × 3 biological replicates) were generated using the NEBNext®Ultra™ RNA Library Prep Kit for Illumina® (NEB, Ipswich, MA, USA) following the manufacturer’s recommendations. Subsequently, adapter ligation was performed, and library quality was assessed on the Agilent Bioanalyzer 2100 system. After cluster generation, the resulting cDNA libraries were paired-end sequenced on an Illumina HiSeqX-10 platform with 150 bp reads generated. The sequencing reads were deposited at the National Center for Biotechnology Information under SRA accession number PRJNA623335 (https://www.ncbi.nlm.nih.gov/bioproject/PRJNA623335/).

### De novo assembly and unigene annotation

The quality of sequencing reads was first estimated using the FastQC toolkit. Then, raw reads were processed to obtain clean reads using Trimmomatic by removing adapters and low-quality reads. Blastall software was used to evaluate the degree of contamination in clean reads, and data filtering was executed in case of contaminants. Clean reads from all samples were pooled together and assembled using Trinity software [48] which combines reads with a certain length of overlap to form longer fragments, termed contigs. Paired-end reads were then mapped back to the contigs, which were connected together to obtain sequences that could not be extended on either end. Such sequences were assembled into unigenes by filtering out redundant sequences. The completeness of the de novo assemblies was assessed using Benchmarking Universal Single-Copy Orthologs (BUSCO) that indicate the number of conserved orthologs present in the transcriptome [49].

To identify the putative functions of assembled *P. cuspidatum* unigenes, a similarity search using BLAST with an e-value cutoff of 1e-5 was performed against publicly available nucleotide and protein databases including Nr, GO, COG, Swiss-Prot, KOG, Pfam, ggNOG, KEGG, and COGs. The Blast2GO program was used to designate GO terms to assembled unigenes under molecular function, biological process, and cellular component categories. Biochemical pathways were assigned to unigenes according to KEGG pathway mapping based on enzyme commission numbers [50].

Coding sequences were predicted using Transdecoder with Pfam domains used as references. TF-encoding transcripts were identified from a similarity search against the Plant Transcription Factor Database based on a Hidden Markov Model profile search.

### Expression levels

Gene expression levels in all samples were estimated by mapping clean reads to the transcripts assembly using the Bowtie program [51], and FPKM values were calculated to measure the expression level of each assembled transcript sequence [52]. Genes expressed in all three tissues were defined as common genes, and those only expressed in a specific tissue were defined as tissue-specific genes. DEGs between two samples were identified by the DESeq2_EBSeq R package. To determine the significance of gene expression differences, we used FDR < 0.01 and |log 2 (fold change)| ≥1 as thresholds. For all comparisons, unigenes with fold-change values > 2 were considered to be up-regulated, whereas those with fold-change values < 2 were down-regulated. DEGs were mapped to GO terms and KEGG pathways and then subjected to an enrichment analysis using a hypergeometric test to find over-represented GO terms and KEGG pathways. The average FPKM value of samples was log-transform normalized for heat map analysis. Each horizontal line of the heat map refers to a gene and the color represents the intensity of gene expression.

### Identification of synthase genes in phenylpropanoid and anthraquinone pathways

Very few biosynthetic genes of phenylpropanoid and anthraquinone pathways in *P. cuspidatum* have been identified. Candidate synthase genes were first identified by KEGG annotation and BLAST search of the *P. cuspidatum* transcriptome using homologous sequences registered in the NCBI as baits. Each selected sequence was further checked by searching the Nr database using BLAST software, and predicted phenylpropanoid and anthraquinone metabolism pathways were drawn by ChemDraw software. A neighbor-joining tree was built by Clustal X 2.0 and MEGA 7.0 with deduced amino acids of polyketide synthase families from the transcriptomes of *P. cuspidatum* and other plants.

### cDNA synthesis and RT-qPCR

Twenty-five DEGs were selected from the *P. cuspidatum* transcriptome for verification of the RNA-seq results (Table S8). In addition to being used for transcriptome sequencing, the remaining RNA was used to synthesize cDNA with the Hifair™ II 1st Strand DNA Synthesis SuperMix (Yeasen, Shanghai, China) according to the manufacturer’s instructions. The primers of all target genes and *ACT* (an internal reference) are listed in Table S8. Experiments were performed on the QuantStudio™ Real-Time PCR system (Applied Biosystems, Waltham, MA, USA) using the Hieff™ qPCR SYBR® Green Master Mix (Yeasen, Shanghai, China). The reaction volume was 10 μL, including 5.0 μL of 2× Hieff™ qPCR SYBR® Green Master Mix, 0.2 μL of each primer (10 μM), 1.0 μL of diluted (1:5) cDNA template, and 3.6 μL of RNase-free water. The thermal cycling profile was: 95 °C for 5 min (hot-start activation) followed by 40 cycles of 95 °C for 10 s (denaturation), 58 °C for 20 s (annealing), and 72 °C for 20 s (extension). Three independent technical replicates were used for each sample and relative expression levels were estimated by the 2^−△△CT^ method [53]. Three replications for each sample were used for real-time PCR analysis. The correlation between qPCR and RNA sequencing data was analyzed using OriginPro 2017 software.

### Extraction and determination of resveratrol and polydatin

To determine the content of resveratrol and polydatin, the root, stem, and leaf of *P. cuspidatum* were extracted as according to the method proposed by Wu et al. [30]. A total of 50 mg of dried fine powdered samples was subjected to ultrasound-assisted extraction by adding 2 mL 80% ethanol (v/v) and holding for 30 min in an ice bath. After centrifuging the extract (12,000 rpm, 4 °C), the supernatant was filtered with a 0.22 μm Nylon filter (Jinlong, Tianjin, China). HPLC analysis was then performed using an Agilent Series 1260 liquid chromatograph (Agilent) with a reverse phase column (SB-C18, 10 μm, 4.6 × 250 mm, Agilent) at room temperature. 0.1% acetic acid in water (solvent A) and acetonitrile (solvent B) were used as the mobile phase with a flow rate of 1 mL/min. The isocratic elution was performed with 26% B for 20 min, then maintained at 95% for 5 min. An injection volume of 10 μL and 305 nm wavelengths were used for detection. Relative retention times were about 4.47 min for polydatin and 11.80 min for resveratrol (Fig. S7). For quantification purposes, standard curve equations were established as follows: polydatin, y = 28,178 × − 24.787 (R^2^ = 0.9999) (Fig. S8A); and resveratrol, y = 52,137 × − 11.948 (R^2^ = 0.9997) (Fig. S8B), where y is the peak area (mAU*s) and × is the content (μg). The contents of resveratrol and polydatin in each tissue were quantified by calculating the peak area compared with those of the respective standards by linear regression. The content was presented as an average percentage of DW from three replicates.

### Extraction and determination of total flavonoids and anthraquinones

Samples (0.1 g) were precisely weighed, then 2.5 mL of 60% ethyl alcohol (v/v) was added, and the samples were vortexed and ultrasonicated in water at 60 °C for 30 min. Supernatant liquor was obtained after centrifuging (12,000 rpm, 10 min), then extracting solution was added to a total of 2.5 mL for subsequent determination. Contents were calculated using a standard calibration curve. The flavonoids content was measured at 502 nm using a UV-visible spectrophotometer according to the instructions given in the plant flavonoids kit (SinoBestBio, Shanghai, China). The standard curve equation was y = 6.2096 × + 0.0008, R^2^ = 0.9996, where y is the absorbance value and × is the content (mg). The anthraquinones content was determined by the magnesium acetate-methanol colorimetry spectrophotometric method using 1, 8-dihydroxy anthraquinone as a standard [54]. The absorbance was detected at 510 nm, and a standard working curve was set up as follows: y = 5.2136 × + 0.0091, R^2^ = 0.9997, where y is the absorbance value and × is the content (mg) (Fig. S8C). All tests were performed in parallel three times. The concentrations of total flavonoids and anthraquinones in the sample were calculated by a linear regression equation.

### Statistical analysis

All data were analyzed using SPSS 20.0 software, and values were expressed as the means ± standard errors of three independent samples. Significant differences (*p* < 0.05) were analyzed using a one-way analysis of variance (ANOVA) and Duncan’s multiple range test.

## Supplementary Information

**Additional file 1:** The unigenes of *Polygonum cuspidatum*.

**Additional file 2: Table S1** The statistical estimation of sequencing data of all samples. **Table S2** Annotation information of unigenes. **Table S3** The mapped pathways of all unigenes of the *Polygonum cuspidatum* transcriptome. **Table S4** FPKM values of major genes involved in stilbene, flavonoid, and anthraquinone biosynthesis pathways identified from the *Polygonum cuspidatum* transcriptome. **Table S5** FPKM values of all unigenes in *Polygonum cuspidatum*. **Table S6** GO enrichment of DEGs. **Table S7** Predicted TFs and their expression in different tissues. **Table S8** 25 DEGs and primers used for RT-qPCR in *Polygonum cuspidatum*.

**Additional file 3: Figure S1** Length distribution of the assembled unigenes of *Polygonum cuspidatum*. **Figure S2** Assessment of assemblies using Benchmarking Universal Single-Copy Orthologs (BUSCO). **Figure S3** Overview of gene expression in *Polygonum cuspidatum*. (A) Boxplot analysis of gene expression profiles in the nine libraries. Boxes represent interquartile ranges; the line across the box represents the median; the plus sign in the box shows mean values; and hyphens over and under the boxes represent the maximum and minimum, respectively. (B) Principal component analysis (PCA) showing clustering pattern among different tissues based on global gene expression profiles. Group1: Root-1, Root-2, and Root-3; Group2: Stem-1, Stem-2, and Stem-3; Group3: Leaf-1, Leaf-2, and Leaf-3. **Figure S4** Phylogenetic tree analysis of polyketide synthase gene families. *CHS* and *STS* identified in this study are indicated by red dots, and the previously reported polyketide synthase genes in *Polygonum cuspidatum* are indicated by blue dots. **Figure S5** The types of differentially expressed TFs. **Figure S6** Heat map analysis of *MYB* and *WRKY* expression in different tissues. **Figure S7** HPLC spectrum of standard substances of resveratrol and polydatin. **Figure S8** Standard curves of (A) polydatin, (B) resveratrol, and (C) anthraquinones.

## Data Availability

The data and materials supporting the conclusions of this study are included within the article and its additional files. The complete raw RNA sequencing datasets generated during the current study are available in the NCBI Sequence Read Archive repository under accession number PRJNA623335 (https://www.ncbi.nlm.nih.gov/bioproject/PRJNA623335/).

## Abbreviations

*P. cuspidatum*: *Polygonum cuspidatum*
PAL: Phenylalanine ammonia-lyase
C4H: Trans-cinnamate 4-hydroxylase
4CL: 4-coumarate-CoA ligase
CHS: Chalcone synthase
CHI: Chalcone isomerase
FNSI: Flavone synthase I
FNS II: Flavone synthase II
F3H: Flavanone3-hydroxylase
F3′5’H: Flavonoid 3′,5′-hydroxylase
F3′H: Flavonoid 3′-hydroxylase
DFR: Dihydroflavonol 4-reductase
FLS: Flavonol synthase
ANS: Anthocyanidin synthase
ANR: Anthocyanidin reductase
STS: Resveratrol synthase
ROMT: Trans-resveratrol di-O-methyltransferase
UGT: Glycosyl transferase
LAR: Leucoanthocyantin reductase
MenF: Isochorismate synthase
MenD: 2-succinyl-5-enolpyruvyl-6-hydroxy-3-cyclohexene-1-carboxylate synthase
MenH: 2-succinyl-6-hydroxy-2,4-cyclohexadiene-1-carboxylate synthase
MenC: O-succinylbenzoate synthase
MenE: O-succinylbenzoic acid-CoA ligase
MenB: Naphthoate synthase
DXS: 1-deoxy-D-xylulose-5-phosphate synthase
DXR: 1-deoxy-D-xylulose-5-phosphate reductoisomerase
ISPD: 2-C-methyl-D-erythritol 4-phosphate cytidylyltransferase
ISPE: 4-diphosphocytidyl-2-C-methyl-D-erythritol kinase
ISPF: 2-C-methyl-D-erythritol 2,4-cyclodiphosphate synthase
HDS: (E)-4-hydroxy-3-methylbut-2-enyl diphosphate synthase
HDR: 4-hydroxy-3-methylbut-2-enyl diphosphate reductase
AACT: Acetyl-CoA C-acetyltransferase
HMGS: Hydroxymethylglutaryl-CoA synthase
HMGR: Hydroxymethylglutaryl-CoA reductase
MVK: Mevalonate kinase
PMVK: Phosphomevalonate kinase
MVD: Diphosphomevalonate decarboxylase
IDI: Isopentenyl-diphosphate delta-isomerase
