# Supplementary material for: Tissue-specific transcriptome analyses reveal candidate genes for stilbene, flavonoid and anthraquinone biosynthesis in the medicinal plant Polygonum cuspidatum
Source: BMC Genomics. 2021 May 17;22:353. doi: 10.1186/s12864-021-07658-3 (PMC8127498; doi:10.1186/s12864-021-07658-3)
Supplement: Supplementary file 3 — Additional file 3: Figure S1 Length distribution of the assembled unigenes of Polygonum cuspidatum. Figure S2 Assessment of assemblies using Benchmarking Universal Single-Copy Orthologs (BUSCO). Figure S3 Overview of gene expression in Polygonum cuspidatum. (A) Boxplot analysis of gene expression profiles in the nine libraries. Boxes represent interquartile ranges; the line across the box represents the median; the plus sign in the box shows mean values; and hyphens over and under the boxes represent the maximum and minimum, respectively. (B) Principal component analysis (PCA) showing clustering pattern among different tissues based on global gene expression profiles. Group1: Root-1, Root-2, and Root-3; Group2: Stem-1, Stem-2, and Stem-3; Group3: Leaf-1, Leaf-2, and Leaf-3. Figure S4 Phylogenetic tree analysis of polyketide synthase gene families. CHS and STS identified in this study are indicated by red dots, and the previously reported polyketide synthase genes in Polygonum cuspidatum are indicated by blue dots. Figure S5 The types of differentially expressed TFs. Figure S6 Heat map analysis of MYB and WRKY expression in different tissues. Figure S7 HPLC spectrum of standard substances of resveratrol and polydatin. Figure S8 Standard curves of (A) polydatin, (B) resveratrol, and (C) anthraquinones. [file 12864_2021_7658_MOESM3_ESM.docx]

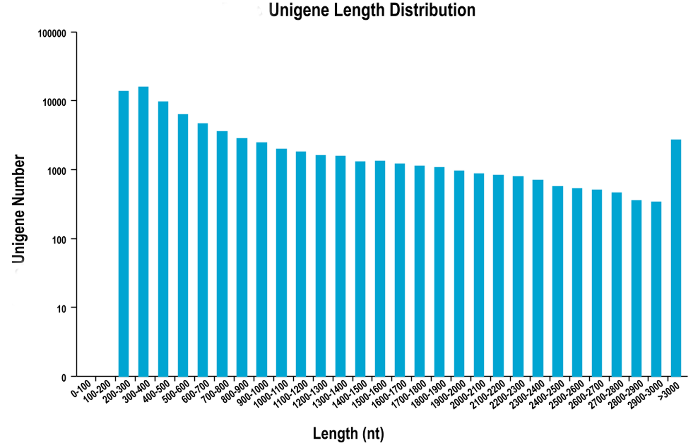


Fig. S1 Length distribution of the assembled unigenes of *Polygonum cuspidatum*.


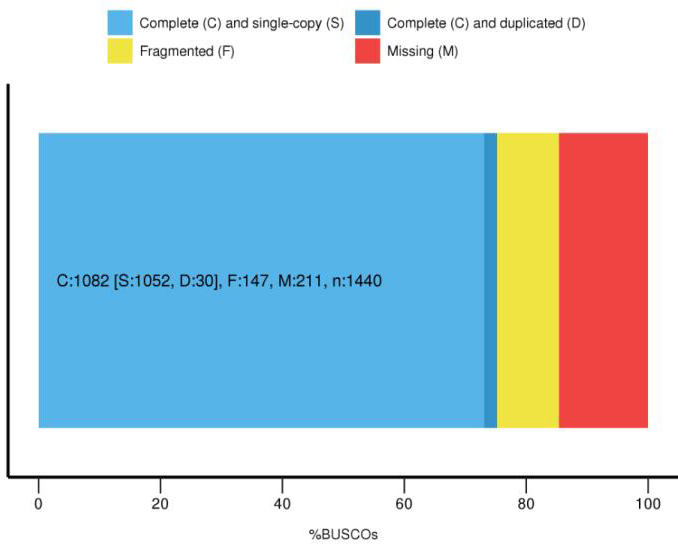


Fig. S2 Assessment of assemblies using Benchmarking Universal Single-Copy Orthologs (BUSCO).


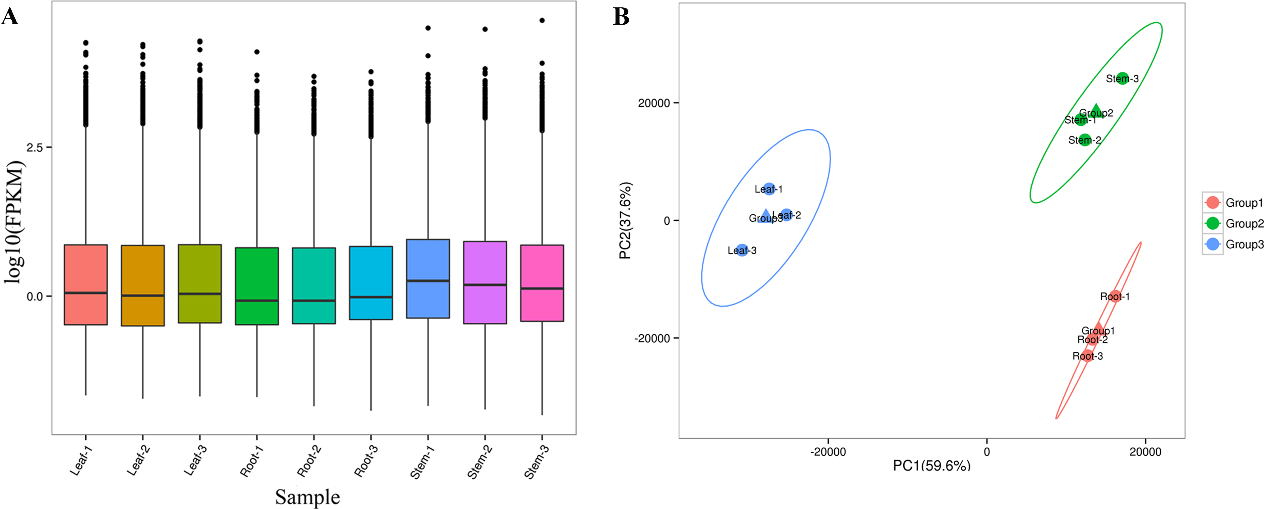


Fig. S3 Overview of gene expression in *Polygonum cuspidatum*. (A) Boxplot analysis of gene expression profiles in the nine libraries. Boxes represent interquartile ranges; the line across the box represents the median; the plus sign in the box shows mean values; and hyphens over and under the boxes represent the maximum and minimum, respectively. (B) Principal component analysis (PCA) showing clustering pattern among different tissues based on global gene expression profiles. Group1: Root-1, Root-2, and Root-3; Group2: Stem-1, Stem-2, and Stem-3; Group3: Leaf-1, Leaf-2, and Leaf-3.
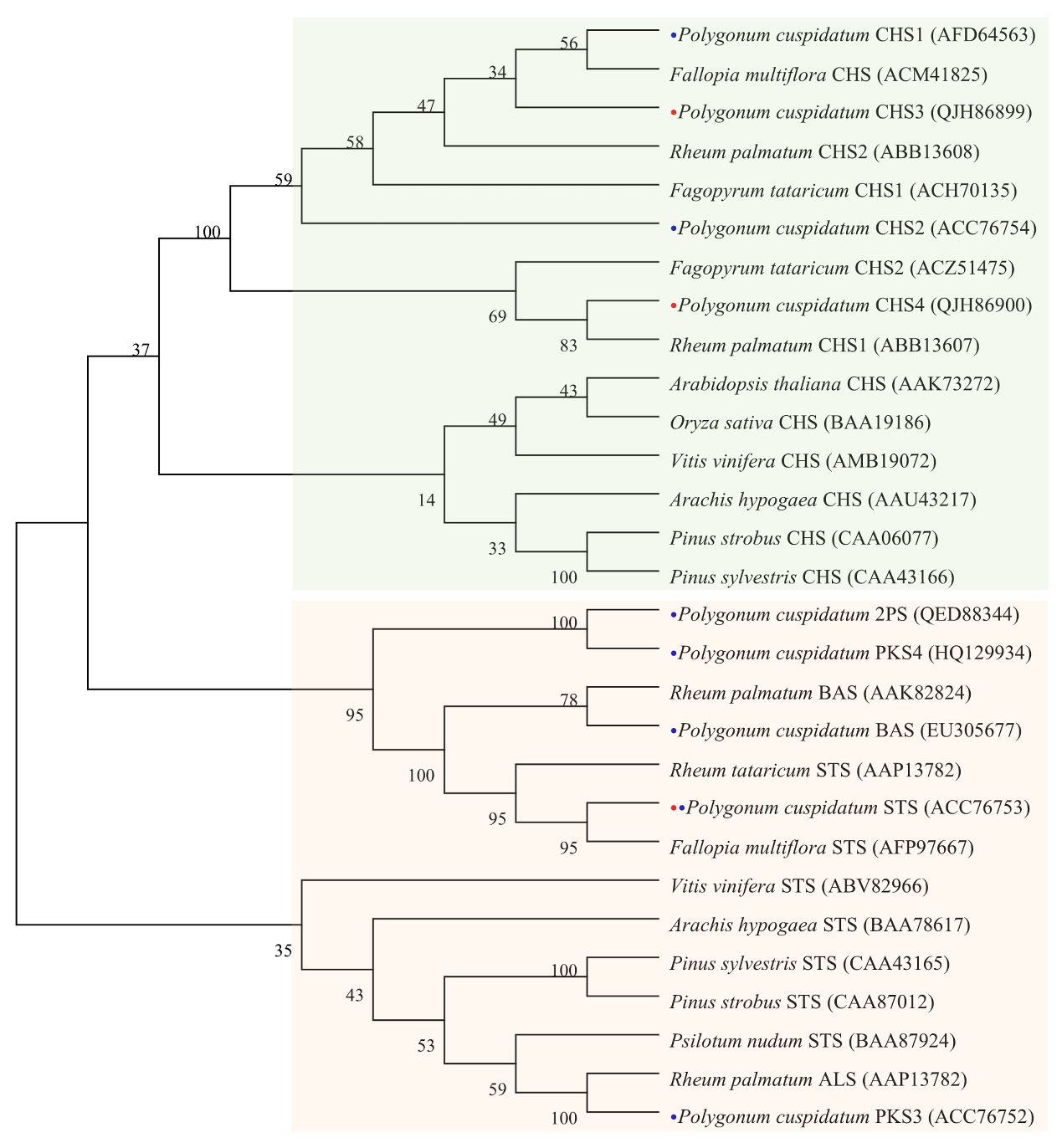


Fig. S4 Phylogenetic tree analysis of polyketide synthase gene families. *CHS* and *STS* identified in this study are indicated by red dots, and the previously reported polyketide synthase genes in *Polygonum cuspidatum* are indicated by blue dots.





Fig. S5 The types of differentially expressed TFs.


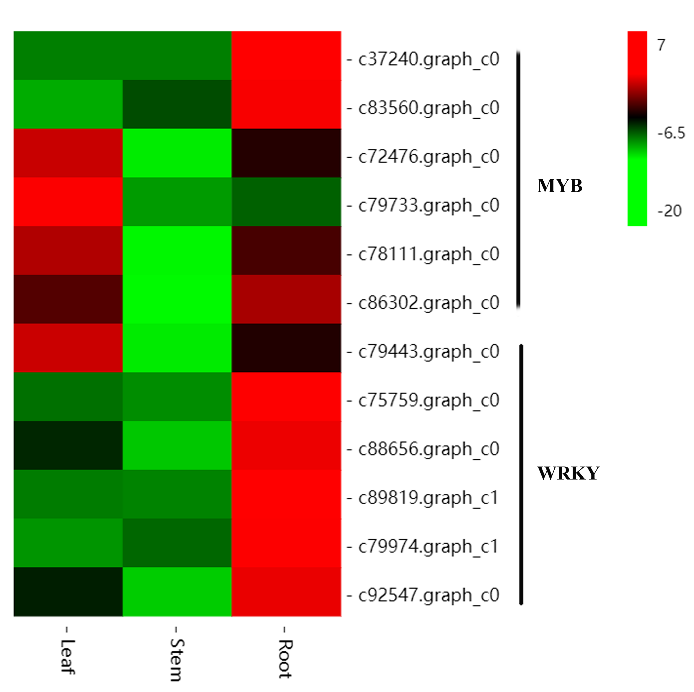


Fig. S6 Heat map analysis of *MYB* and *WRKY* expression in different tissues.


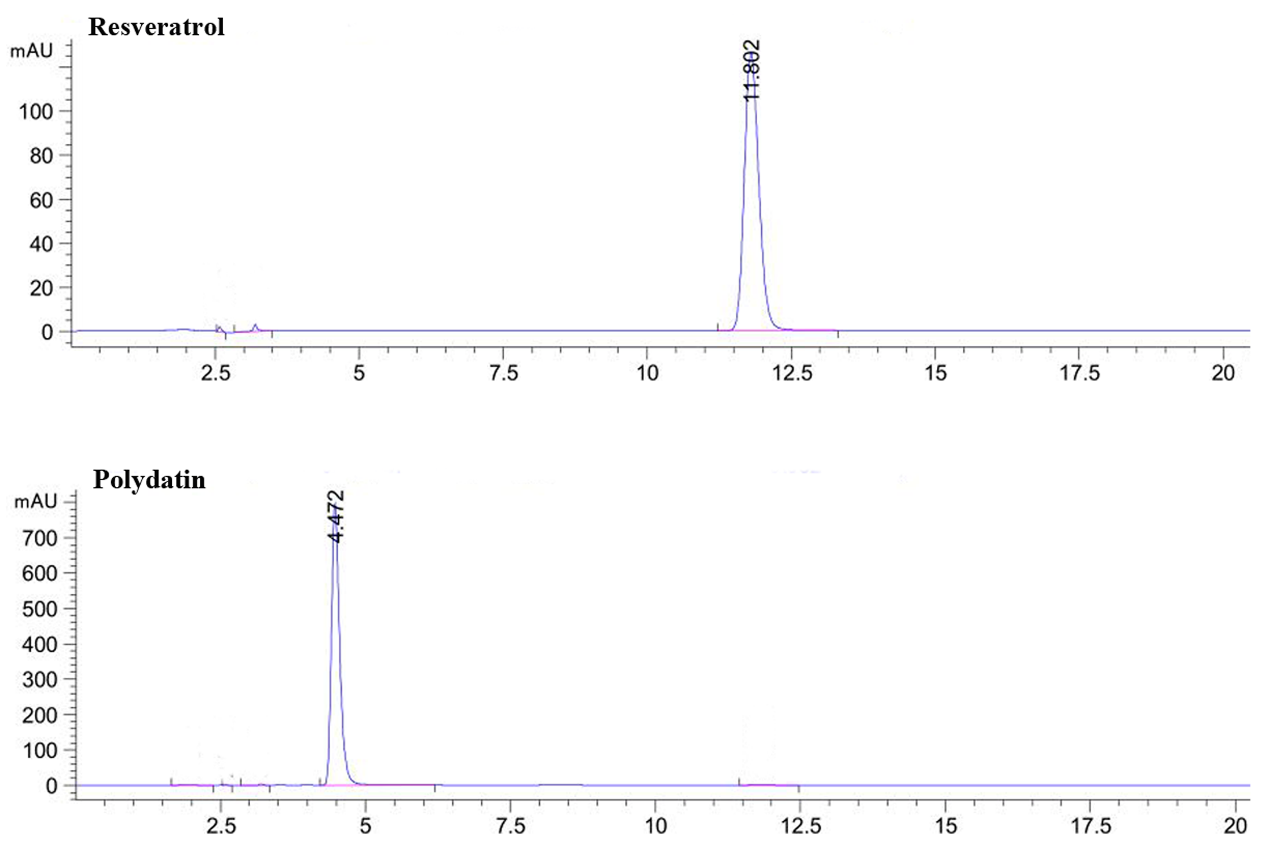


Fig. S7 HPLC spectrum of standard substances of resveratrol and polydatin.


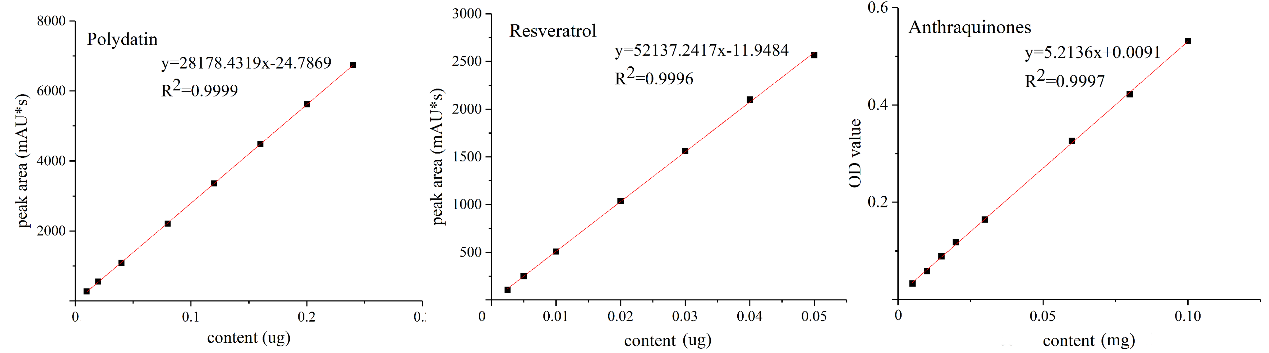


Fig. S8 Standard curves of (A) polydatin, (B) resveratrol, and (C) anthraquinones.
